# Natural Bioactive Compounds in the Management of Oral Diseases in Nephropathic Patients

**DOI:** 10.3390/ijerph19031665

**Published:** 2022-01-31

**Authors:** Michele Basilicata, Manuela Di Lauro, Vincenzo Campolattano, Giulia Marrone, Roberto Celotto, Anna Paola Mitterhofer, Patrizio Bollero, Nicola Di Daniele, Annalisa Noce

**Affiliations:** 1 UOSD Special Care Dentistry, Department of Experimental Medicine and Surgery, University of Rome Tor Vergata, 00100 Rome, Italy; michele.basilicata@ptvonline.it; 2 UOC of Internal Medicine-Center of Hypertension and Nephrology Unit, Department of Systems Medicine, University of Rome Tor Vergata, 00133 Rome, Italy; giulia.marrone@uniroma2.it (G.M.); didaniele@med.uniroma2.it (N.D.D.); 3 UOSD Special Care Dentistry, Department of Dentistry and Dental Prosthesis, University of Rome Tor Vergata, 00133 Rome, Italy; vincenzo.campolattano@students.uniroma2.eu; 4 Department of Cardiovascular Disease, University of Rome Tor Vergata, 00133 Rome, Italy; Roberto.celotto@alumni.uniroma2.eu; 5 Nephrology and Dialysis Unit, Department of Systems Medicine, University of Rome Tor Vergata, 00133 Rome, Italy; annapaola.mitter@uniroma2.it; 6 UOSD Special Care Dentistry, Department of Systems Medicine, University of Rome Tor Vergata, 00100 Rome, Italy; patrizio.bollero@ptvonline.it

**Keywords:** chronic kidney disease, chronic periodontitis diseases, polyphenols, oral microbiota, renal replacement therapy, special care dentistry

## Abstract

Among the chronic non-communicable degenerative diseases (CDNCDs), chronic kidney disease (CKD) represents a global public health problem. Recent studies demonstrate a mutual cause–effect relationship between CKD and oral diseases, in which the presence of one induces the onset and faster progression of the other. In particular, the oral cavity alterations more frequent in CKD patients are: chronic periodontitis diseases, bone lesions, oral infections, and oral cancer lesions. Currently, a standardized therapy for the treatment of oral diseases is lacking. For this reason, natural bioactive compounds (NBCs), characterized by several health effects, such as antioxidant, antimicrobial, anti-inflammatory and anti-cancer actions, represent a new possible adjuvant therapy in the management of these pathological conditions. Among NBCs, polyphenols play a leading role due to positive modulation of oral microbiota, preventing and correcting oral dysbiosis. Moreover, these compounds exert anti-inflammatory effects, such as inhibiting the production of pro-inflammatory cytokines and the expression of cycloxigenase-2. In this light, the formulation of a new mouthwash/gel/gingival paste, with a high content of polyphenols in association with NBCs characterized by antimicrobial action, could represent a future therapy of oral disease in CKD patients.

## 1. Introduction

Oral diseases are among the most widespread pathologies in the world and have rapid diffusion, becoming a real global epidemic in the last few years. Oral diseases are often associated with debilitating conditions that reduce the quality of life of patients and increase public health costs [1]. For this reason, over the years greater attention has been paid to oral preventive education, encouraging the use of good hygiene practices, and discouraging poor lifestyle habits, such as smoking, alcohol abuse and excessive consumption of sugar [2,3]. Oral health appears to be closely related to an individual’s overall health [4]. In fact, the onset of an oral disease, for example caries or periodontitis, seems to represent an important risk factor for the development of several chronic non-communicable degenerative diseases (CDNCDs), including chronic kidney disease (CKD) [5,6]. Several studies suggest that chronic periodontitis disease (CPD) is associated with an increased risk of diabetes mellitus, arterial hypertension, atherosclerosis and neurodegenerative diseases [7,8,9,10,11,12,13]. The oral diseases and CKD seem to be connected to each other in a mutual cause–effect relationship, in which the presence of one induces the onset and progression of the other [14,15]. Recent epidemiological studies suggest that CKD patients appear to be more predisposed to oral diseases compared to the general population [16,17]. However, the mechanisms underlying this relationship are still poorly studied and the available data need to be confirmed by larger randomized clinical trials. The possible link between the two diseases could be represented by the alteration of the oral microbiota, a set of symbiotic bacteria in the oral cavity [18]. In fact, on the one hand, oral diseases induce an alteration of the oral microbiota, which loses its ability to protect the host from pathogenic bacteria that can enter through the buccal opening. For this reason, the alteration of oral microbiota composition could represent a risk factor for the CDNCDs onset. Therefore, these systemic diseases could induce, in turn, an alteration of the oral microbiota that is associated with an increased susceptibility to the oral disease onset [19,20]. Currently, one of the main topics of research is focusing on the use of strategies to prevent alterations of the oral microbiota and to safeguard the host’s general health [18]. In this context, the use of natural products which, free from side effects, act by counteracting the occurrence of an oral microbiota dysbiosis, protecting from the CDNCDs, could represent a valid adjuvant strategy [21].

Natural bioactive compounds (NBCs) are biological molecules of food origin, mainly contained in plant-based food, which exert important beneficial effects in the human body [22,23]. The NBCs contained in plant-based food are called phytochemicals and they can be classified into different categories, such as polyphenols, carotenoids, alkaloids, phytosterols, nitrogen and organosulfur compounds [24]. Several in vitro and in vivo studies show that NBCs seem to exert important anti-inflammatory, antioxidant, antimicrobial, anticancer, cardioprotective, neuroprotective, hepatoprotective, hypoglycaemic and antihypertensive effects [25,26,27,28]. Furthermore, NBCs are able to positively modulate the oral microbiota composition [18]. Consequently, the intake of NBCs could represent a valid strategy able to counteract the onset and the progression of CDNCDs, including CKD. In this regard, the purpose of this review is to define the relationship between oral diseases and CKD and to analyze the possible role of NBCs consumption in the prevention and treatment of oral diseases. In particular, among the oral diseases, in this review we examine oral infections, dental and oral bone tissue alterations, saliva and exhaled modifications, and oral soft tissue negative changes.

## 2. Research Methods

To reach the article purpose, we selected a list of studies according to research online items of “natural bioactive compounds” and “oral disease” and “chronic kidney disease” and “periodontitis” in association with “epigallocatechin gallate” and/or “polyphenols” and/or “oral microbiota” and/or “oral inflammation” and/or “renal replacement therapy” and/or “haemodialysis” and/or “peritoneal dialysis” and/or “kidney transplant” and/or “xerostomia”. The databases used were PubMed and Web of Science up to January 2022. Studies were all in English language and they were manually retrieved by the authors.

## 3. Oral Health and Chronic Kidney Disease (CKD)

**Figure 1:**
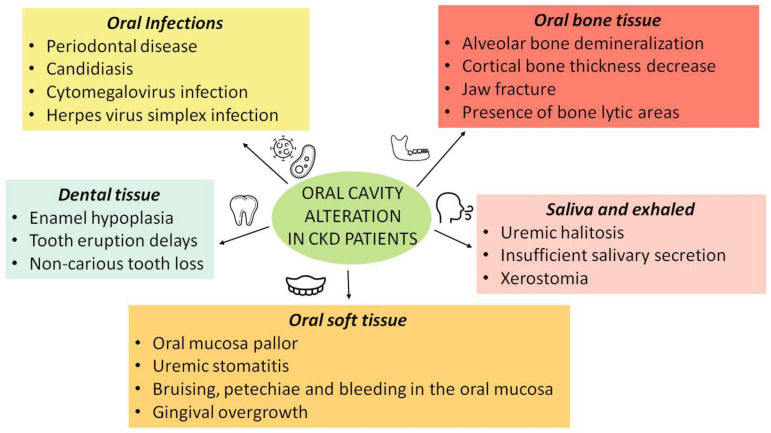
Oral cavity alteration in chromic kidney disease (CKD) patients.

Oral health of CKD patients is significantly compromised, and it is estimated that nearly 90% of them present an oral cavity alteration, including periodontal disease, impaired mineralization of the bone matrix, gingival hyperplasia and alterations of saliva secretion and composition [29]. Furthermore, CKD patients show a greater predisposition to oral infections [30]. These changes may be related both to CKD itself and to the effect of renal replacement therapy (RRT) [31,32]. At the same time, poor oral hygiene care can favor and exacerbate the chronic low-grade inflammatory state, typical of CKD [33]. In order to prevent oral diseases in CKD patients, it is necessary to develop specific oral hygiene strategies. Moreover, it has been shown that the presence of a lesion or other pathological processes of the oral cavity, if not treated, can accelerate the progression or induce the onset of CKD comorbidities [31]. Oral diseases and CKD appear to have a bidirectional correlation with a reciprocal cause–effect relationship (Figure 1) [34].

### 3.1. Effects of CKD Comorbidities on Oral Health

Some of the main uremic comorbidities may represent important risk factors for the oral alterations onset, including uremic toxins accumulation, normochromic and normocytic anemia, water–electrolyte imbalance, calcium-phosphorus metabolism alteration and possible concomitant malnutrition [35].

#### 3.1.1. Saliva and Exhaled Alteration

Nearly one third of end stage renal disease (ESRD) patients show a condition known as uremic halitosis [36]. In fact, the loss of renal function, especially in more advanced CKD stages, induces urea accumulation both in saliva and serum. In the oral cavity, excess urea is converted into ammonia by the urease–positive microbial flora, responsible for the typical halitosis of ESRD patients [35]. Several studies have shown a direct correlation between the serum accumulation of urea, and other uremic toxins, with the present of ammonia in CKD patients’ exhalation [37]. The ammonia accumulation in the oral cavity is responsible for the reduction in salivary flow and for the mouth dry, phenomena usually observed in ESRD patients [36]. Furthermore, this condition leads to the perception of a metallic and unpleasant taste following the ingestion of food, which induces the patient to reduce caloric and protein intake, contributing to the development of protein-energy wasting (PEW) syndrome, typical of CKD patients [38]. In fact, CKD patients present often this syndrome which is characterized by muscle hypercatabolism, chronic inflammatory state and metabolic acidosis [38,39,40]. This condition causes non-intentional reduction of body weight and loss of muscle mass, with a concomitant increase of pro-inflammatory cytokines, such as tumor necrosis factor (TNF)-α and interleukin (IL)-6, partly responsible for the onset of a chronic inflammatory state [41]. In addition, the pro-inflammatory state tends to decrease the albumin in the circulation, exacerbating the state of malnutrition [42]. The establishment and the worsening of PEW syndrome increase the hospitalization rate and all-causes mortality of CKD patients [38]. The loss of muscle mass leads to a further increase in urea levels, in the blood and the saliva, enhancing uremic halitosis and leading to the development of a vicious circle [35]. In addition to serum urea high level, other factors probably implicated in the onset of uremic halitosis are the increase in the serum phosphorus concentration and the alteration of salivary pH [43,44,45]. Uremic halitosis can also be associated with a burning sensation on the lips and tongue by neuropathic origin [46] or even with a sensation of an enlarged tongue [44].

#### 3.1.2. Oral Soft Tissue Alterations

Other oral abnormalities often found in CKD patients concern oral soft tissue. Often CKD patients in more advanced stages present a normochromic and normocytic anemia, mainly due to a deficit in the production of erythropoietin and to a reduction in the half-life of red blood cells. The anemia is at the basis of the characteristic pallor of the mucous membranes which is often observed in CKD patients [47].

Moreover, a clinical condition known as uremic stomatitis may be encountered in ESRD patients. This is an oral complication of unknown etiology which is relatively uncommon [43,46,48]. Clinically, uremic stomatitis is characterized by the presence of erythematous lesions in the oral cavity, which can be localized or generalized. These lesions are covered with a pseudomembranous exudate that can be removed, leaving an intact or ulcerated mucosa [45]. Uremic stomatitis is determined by lesions that often heal spontaneously, but in order to promote their healing, the treatment with a mildly acidic rinse, such as 10% hydrogen peroxide, appears to be effective [43].

#### 3.1.3. Dental Tissue Alterations

In the presence of CKD, it is possible to observe anomalies in the teeth. A sign frequently encountered in CKD patients is enamel hypoplasia. It mainly associated with the alteration of calcium-phosphorus metabolism [48]. Hypoplasia of the dental enamel is a pathological condition characterized by the lack of development of the dental enamel, which therefore presents a quantitative deficit and a reduced thickness. A patient suffering from enamel hypoplasia experiences problems not only of an aesthetic nature, but this pathological condition can also represent an important risk factor for the development of more serious oral diseases [47]. Dental enamel health is deeply affected by a patient’s nutritional status. Vitamin D, calcium, vitamin A deficiency and the present of PEW have been associated with enamel hypoplasia, conditions which also lead to increased susceptibility to tooth decay [49]. Enamel hypoplasia is a condition that is often found in CKD pediatric patients. In fact, a prolonged deficiency of vitamin A, vitamin D and calcium during the development of the teeth can lead to an enamel atrophy and a dentin defective apposition and calcification [49]. In particular, Bawden et al. hypothesized that a hypovitaminosis D leads to an inadequate transport of calcium, useful for the dental tissues’ development [50]. Vitamin D deficiency affects the structure of the teeth, and it delays their physiological eruption. In the presence of vitamin D deficiency, the teeth are microscopically characterized by an extended layer of predentin, by the presence of interglobular dentin, and by enamel imbalance formation [51]. In CKD adult patients, on the other hand, hypovitaminosis D can lead to the narrowing or the calcification of the pulp chamber [35].

Currently, there is no unambiguous consensus regarding the increased risk of developing dental caries in CKD patients [44]. A potential antibacterial effect was, in fact, attributed to the increase in salivary pH, due to the hydrolyzation of urea by saliva, suggesting its protective action against caries [47]. In contrast, non-carious tooth loss is more prevalent in CKD patients than in the general population. This loss can be induced by uremic gastritis and by gastro-esophageal reflux, which often occur in ESRD patients, and which can be responsible for tooth erosion [35].

#### 3.1.4. Oral Bone Tissue Alteration

In CKD patients, further alterations of the oral cavity are linked to the alteration of calcium–phosphorus metabolism, vitamin D abnormal metabolism and compensatory hyperplasia of parathyroid, causing the development of CKD-mineral and bone disorder (CKD-MBD) [35]. At the oral cavity level, CKD-MBD is characterized by demineralization of the alveolar bone, reduction of trabecular bone, decrease of cortical bone thickness, metastatic calcifications of soft tissues, fibrocystic lesions, lytic bone areas, fracture of the jaw (spontaneous or after dental procedures), by abnormal bone healing after an extraction and, sometimes, by tooth mobility secondary to bone substance loss [47].

### 3.2. Effects of Renal Replacement Therapy on Oral Health

In addition to oral changes due to comorbidities related to CKD and CKD itself, other anomalies in the oral cavity can be induced by RRTs, such as dialysis (hemodialysis-HD and peritoneal dialysis) and kidney transplant.

#### 3.2.1. Oral Alteration in Dialysis Patients

In HD patients, the bleeding of the oral mucosa is frequent. It may be due to CKD itself, such as platelet alteration functions and normochromic and normocytic anemia [52,53], or HD treatment. The latter can induce or exacerbate the condition of thrombocytopenia caused by mechanical damage and heparin use during HD procedure. For these reasons, HD predisposes patients to bruising, petechiae and bleeding in the oral mucosa [45].

Furthermore, due to the reduced water intake and polypharmacy, a condition of xerostomia is often found in HD and peritoneal dialysis patients [44]. Xerostomia is characterized by dryness of the oral cavity resulting from insufficient salivary secretion or from a complete lack of saliva. Based on its pathogenesis, it is classified as true xerostomia (primary xerostomia), resulting from malfunction of the salivary glands, or pseudo-xerostomia, also known as symptomatic xerostomia (xerostomia spuria), during which the patient has a subjective impression of a dry mouth despite the normal function of the salivary glands [54]. Xerostomia can affect oral function and can compromise the patient’s well-being. In fact, salivary secretions are vital for oral health, as they are involved in mechanical cleaning with a protective function. Hyposalivation can also increase the risk of oral infections, such as candidiasis, and the patient’s susceptibility to dental caries, periodontal disease and tooth loss [44].

In HD patients, epidemiological studies show that oral hygiene is usually poor so the deposits of tartar and plaque can be increased [35].

Currently, few data are present in literature about the relationship between peritoneal dialysis and CPD.

#### 3.2.2. Oral Alteration in Renal Transplant Patients

Renal transplant patients often undergo immunosuppressive therapy which makes them more susceptible to infections and to the development of malignancies [55]. Renal transplant patients are, therefore, more vulnerable to fungal infections, including *Candida albicans*, which can induce lesions of the oral mucosa and the perioral [35]. One of the main complications of fungal infections is angular cheilitis, which is an inflammatory disorder that affects the corners of the mouth. Angular cheilitis is observed in more than 4% of renal transplant patients. Other forms of candidiasis have been reported in renal transplant patients, including pseudomembranous (1.9%), erythematous (3.8%), chronic atrophic—also called prosthetic stomatitis (3.8%) [35].

Among viral infections, cytomegalovirus (CMV) and herpes virus simplex (HSV) are frequently associated with the use of immunosuppressive therapy. In particular, the ulceration of the oral mucosa is often associated with CMV infection, with a preference for the lateral edges of the tongue [55].

In addition, a secondary effect of cyclosporine therapy, known as gingival overgrowth (GO), may be observed in renal transplant patients. GO secondary to immunosuppressive therapy is the most studied oral alteration in renal transplant patients. It has been observed that if patients are treated with a combination of cyclosporine and nifedipine, the prevalence of GO increases to 50%. A study by Marshall et al. highlighted how this effect occurs within 3 months from the beginning of this pharmacological treatment. This overgrowth, which normally begins at the interdental papillae, is most common in the anterior segments of the mouth and on the labial surfaces of the teeth [56]. GO does not seem to have a predilection for the maxilla or the mandible [35]. Given the numerous side effects of cyclosporine affecting the oral cavity, other therapeutic alternatives have been developed. In fact, tacrolimus, rapamycin, and mycophenolate mofetil are used to replace cyclosporine. These therapeutic alternatives are characterized by a GO decrease. However, these drugs are more expensive, and all their side effects are not yet known. For these reasons, in many cases, cyclosporine remains the first immunosuppressive therapeutic option [35].

Cases of malignant lesions, such as squamous-cell carcinoma and Kaposi sarcoma, which could develop in GO areas induced by cyclosporine, have also been described in renal transplant patients. The increased risk of malignant lesions in the oral cavity in renal transplant patients is a direct consequence of long-term immunosuppressive therapy [44].

### 3.3. Periodontal Disease and CKD

CPD is an infectious disease caused by Gram-negative bacteria responsible for destruction of the supporting tissues of the teeth. The presence of these bacteria in the subgingival biofilm causes the release of proteolytic enzymes, capable of degrading the gingival tissue [57]. Periodontal pathogens not only induce inflammation and local destruction of oral tissues, but they are also associated with the onset of a systemic inflammatory state. An interesting study conducted on 359 CKD patients highlighted how non-surgical periodontal treatment induced a reduction in systemic inflammation, monitored by pro-inflammatory cytokines [58]. Therefore, it is hypothesized that the treatment of CPD in CKD patients may have a positive effect on oxidative stress (OS) and on systemic inflammatory state [16,59]. These last represent prognostic factors associated with more sudden worsening of residual renal function [60]. Therefore, it is plausible to hypothesize that CPD may represent an important risk factor for the onset and the progression of CKD [58]. Furthermore, the mortality rate of CKD patients seems to be profoundly increased in the presence of periodontitis [16].

The microbial complexes in the subgingival biofilm during CPD were classified according to their pathogenic potential into five groups: red, green, orange, yellow and purple [61]. Notably, the red group, which is composed of *Tannerella forsythia*, *Treponema denticola*, and *Porphyromonas gingivalis*, has been identified as the major and severe cause of CPD [61]. These phenomena seem to be related to the dysbiosis of the oral microbiota characterized by increased microbial diversity and by the presence of anaerobic bacterial species [62,63], highlighting new potential pathogenic species associated with CPD, such as *Filifactor alocis*, *Fretibacterium fastidiosum* and *Treponema vincentii* [64,65]. Furthermore, red complex bacteria and *Candida albicans* are present more frequently in CKD patients with CPD than in healthy individuals [58]. Therefore, it is essential to perform a periodic examination of the oral cavity in CKD patients to allow an early diagnosis and a prompt treatment of CPD [57].

Some of these periodontal-pathogenic microorganisms play a direct action on the progression of CKD. In particular, periodontal bacteria can compromise the kidney healthiness through several mechanisms: (i) indirect, such as the systemic bacteremia, which develops from gingival ulcerations and which allows pathogenic microorganisms to enter the bloodstream, and the release of pro-inflammatory cytokines, responsible for tissue destruction; (ii) direct, such as the destruction of the alveolar bone [59].

During periodontal inflammation, the interaction between the pathogenic microorganism and the immune system leads to the secretion of pro-inflammatory cytokines, which in turn attract other cells of the immune system, more specific against the recognized pathogen, amplifying the inflammatory state. Cytokines are secreted by different cell groups and can act both as inflammation amplifiers and as responsible of direct tissue destruction. Inflammatory cytokines enhance vascular permeability, which in turn can increase bacteremia and stimulate fibroblasts and inflammatory cells, inducing the release of other cytokines. During inflammation, the expression of endothelial adhesion molecules also increases, such as the intercellular adhesion molecule (ICAM)-1 and the vascular cell adhesion molecule (VCAM)-1, E-selectin and chemokines (monocyte chemoattractant protein-1 and IL-8) [66,67].

Among other typical features associated with CPD, OS plays a pivotal role in the relationship between periodontitis and CKD. In CPD, reactive oxygen species (ROS) are an important primary defense system, and they are produced by inflammatory cells [68]. It is plausible that OS has a significant impact on local periodontal lesions. The effects of OS on systemic inflammation have been demonstrated by several research groups. For example, tissue levels of 8-hydroxydeoxyguanosine (8-OHdG), an OS marker, are increased in multiple organs, such as liver, heart, kidneys, and brain in an animal model of periodontal inflammation [69]. Therefore, it is hypothesized that the balance between pro-oxidant and antioxidant species is associated with the onset, the severity and the progression of CPD and its systemic complications [59].

## 4. Use of Natural Bioactive Compounds (NBCs) to Prevent and Treat Oral Diseases in CKD Patients

**Figure 2:**
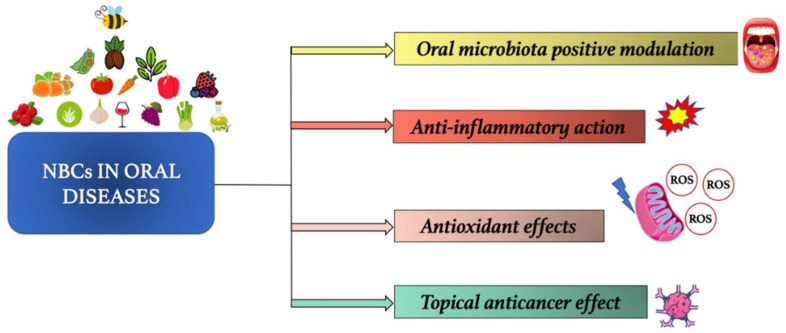
Natural bioactive compounds (NBCs) main actions in oral diseases.

Numerous studies suggest that NBCs exert important beneficial effects in safeguarding oral cavity health [18]. The NBCs are organic compounds present in nature and they are able to exert healthy actions. These compounds are contained in numerous food sources, mostly in plant-based ones, such as fruit and vegetables [28]. Recently, foods rich in NBCs have been characterized for their beneficial proprieties and for this reason they are called “functional foods”. To date, more than 5000 NBCs have been isolated from plant-based foods and they have been classified according to their chemical structure in phenolics, carotenoids, alkaloids, phytosterols, nitrogen-containing compounds, and organosulfur compounds [70]. Phenolic compounds, especially polyphenols, are undoubtedly the most studied. The beneficial effects exerted in the oral cavity can be displayed both using NBC-based products, commonly employed in oral hygienic practice, such as toothpastes and mouthwashes, and through the NBCs oral administration, as food or as dietary supplements. In the first case, NBCs seem to exert a direct action through contact with the cells and tissues of the oral cavity, displaying their effects without the loss of efficacy due to a lack of systemic distribution or to the reduction of their bioavailability, which is observed when the NBCs are assumed through foods or oral food supplements [71,72] (Figure 2).

**Figure 3:**
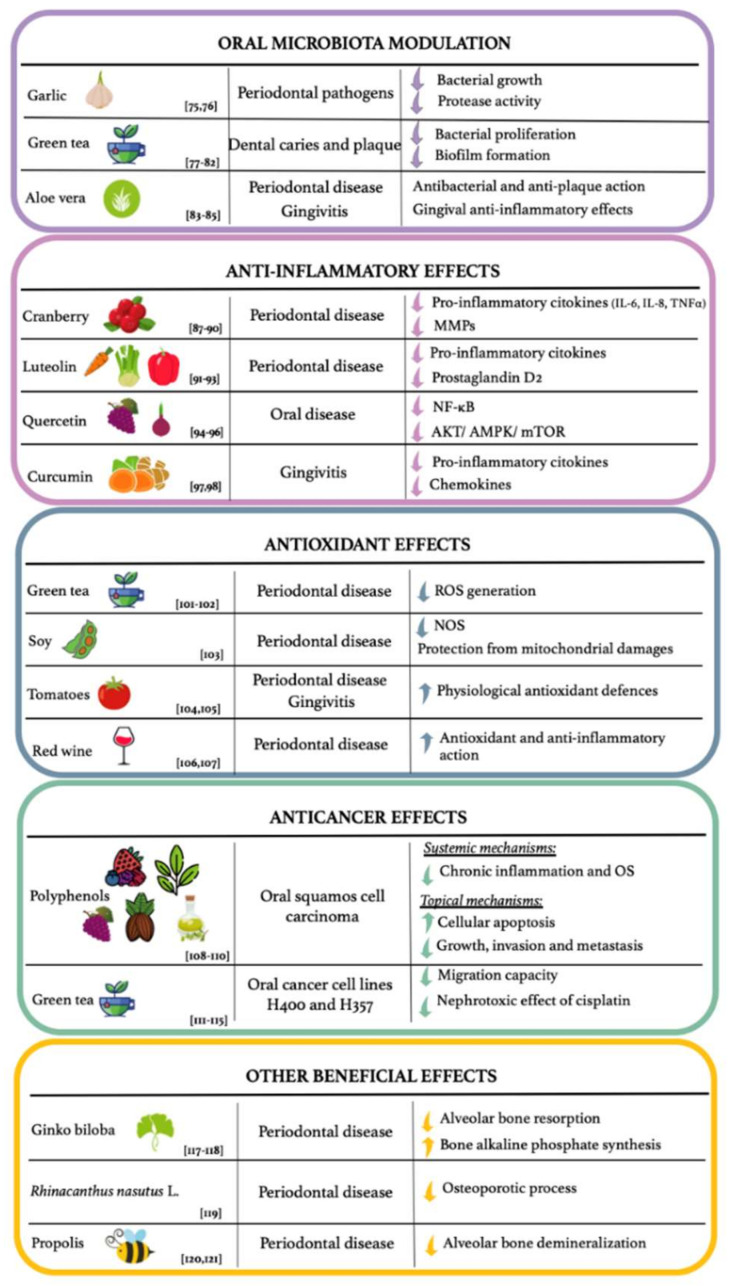
Possible beneficial effects of natural bioactive compounds on oral diseases in chronic kidney disease patients. Abbreviations: AKT, protein kinase B; AMPK, AMP-activated protein kinase; IL-6, interleukin-6; IL-8, interleukin-8; MMPs, matrix metalloproteinases; mTOR, mammalian target of rapamycin; NF-κB, nuclear factor-κB; NOS, nitric oxide synthase; OS, oxidative stress; ROS, reactive oxygen species; TNF-α, tumor necrosis factor α.

The NBCs act thanks to various mechanisms in countering the oral diseases onset and progression: (i) they can act by the oral microbiota positive modulation and thus, preventing the onset of dysbiosis, closely related to the increase in oral infections susceptibility; (ii) they are able to exert an anti-inflammatory action, preventing/treating inflammatory oral diseases, such as periodontal disease; (iii) they perform antioxidant effects, preventing the OS. The latter impairs the immune defences, making the host more susceptible to oral infectious diseases, such as gingivitis by herpes virus and candida; (iv) they exert also a topical anticancer effect on oral cavity cells and tissues, reducing the onset and progression of cancer typical of the oral cavity, such as oral squamous cell carcinoma (Figure 3).

### 4.1. Modulation of the Oral Microbiota

Several studies highlight how NBCs are able to positively modulate the oral microbiota, preventing oral dysbiosis, usually observed in CKD patients (Figure 3) [73].

Garlic has well-known antimicrobial and antifungal proprieties, thanks to its content in allicin, an organosulfur compound that is able to inhibit the proliferation of various oral pathogens, including *Porphyromonas gingivalis* and *Aggregatibacter actinomycetemcomitans* [74]. In particular, allicin appears to inhibit the bacterial growth and protease activity of periodontal pathogens, suggesting how the extracts of *Allium sativum* could represent a valid therapeutic strategy in the management of oral infections [75]. 

Green tea extract, rich in polyphenols, seems to counteract the proliferation of the most common bacteria that induce dental caries, including *Streptococcus mutans* and *Streptococcus salivarius* [76]. Green tea extract exerts a bactericidal effect through various mechanisms, such as damage and lysis of bacterial cell membranes, inhibition of ATP-synthase and of bacterial energy metabolism and prevention of the microorganism adhesion on the oral surface. All these mechanisms ensure that pathogenic bacterium is no longer able to colonize the host [77,78].

The catechins and epigallocatechin of cocoa (*Theobroma cacao*) seem to have important anti-cariogenic properties, inhibiting the biofilm formation and proliferation of the main oral pathogens that induce caries and plaque, such as *Streptococcus mutans* and *Streptococcus sanguinis* [79,80]. In particular, cocoa polyphenols inhibit the bacterial production of sucrose by enzyme dextransucrase, responsible for the formation of plaque [81].

Aloe vera is a further powerful oral antimicrobial and antiviral agent, thanks to the presence of an active compound named aloin, anthaquinone compound [82]. Aloe vera would seem to exert anti-plaque and gingival anti-inflammatory effects similar to that of chlorhexidine, a broad-spectrum antibacterial agent, but totally free of side effects [83]. In fact, aloe vera exerts its anti-inflammatory proprieties inhibiting cyclooxygenase (COX) pathway and reducing prostaglandin E2 production from arachidonic acid [84].

### 4.2. Anti-Inflammatory Effects

NBCs exert important anti-inflammatory effects in the oral cavity [85]. In CKD patients, NBCs could be used in the prevention or in the treatment of inflammatory oral diseases (Figure 2).

The cranberry proanthocyanins seem to exert anti-inflammatory effects in the oral cavity, through different mechanisms. They appear to be able to inhibit the production of pro-inflammatory cytokines (such as IL-6, IL-8 and TNF-α) by gingival fibroblasts, the main cells mediating the inflammatory response of the periodontal tissues [86]. Furthermore, in vitro studies suggest that cranberry proanthocyanins inhibit the expression of COX-2, responsible for the prostaglandin E2 production (important inflammatory mediator) [87]. Finally, cranberry proanthocyanins inhibit both the activity and the synthesis of matrix metalloproteinases (MMPs), enzymes produced by inflammatory cells in response to periodontopathogens. Moreover, these enzymes are involved in the degradation of periodontal tissue and alveolar bone resorption, phenomena observed in periodontal disease [88]. For this purpose, the use of cranberry-based toothpaste or oral supplements could represent a valid treatment to counteract inflammatory oral diseases, such as periodontitis [89].

Another NBC with proven anti-inflammatory effects is luteolin, a flavone contained mainly in carrots, fennel, and pepper. In vitro and in vivo studies have shown that luteolin inhibits the production of IL-6, TNF-α and prostaglandin D2 by gingival fibroblasts, if they are stimulated by periodontopathogens, and it inhibits the expression of COX-2 [90,91,92].

Furthermore, in vitro studies show that quercetin, a flavonoid in grapes, apples, and onions, inhibits the synthesis of TNF-α by oral macrophages through the nuclear factor-κB (NF-κB) pathway suppression [93,94]. Moreover, quercetin seems to reduce the IL-1β, IL-6, IL-8 and TNF-ɑ production and the COX-2 activity, suppressing the AKT/AMPK/mTOR pathway, in oral mucosal keratinocytes. These beneficial actions demonstrate quercetin’s protective role in countering inflammatory oral diseases [95].

Curcumin, the main polyphenol in turmeric, is often used as a natural remedy in the treatment of periodontal diseases due to its anti-inflammatory power, therefore in the market there are several curcumin-based toothpastes and mouthwashes [96]. Curcumin appears to reduce the release of inflammatory prostaglandins, IL-1β and chemokine ligand 28 (CCL28) levels, in the gingival crevicular fluid in patients with gingivitis, after topical administration of a curcumin-based gel [97].

### 4.3. Antioxidant Effects

Among the beneficial effects of NBCs there are antioxidant actions, that are exerted both by stimulating the natural antioxidant defence mechanisms and by directly neutralizing the pro-oxidant species. Several studies have highlighted how NBCs counteract ROS overproduction, which can be observed in patients with periodontitis and with CKD (Figure 2) [98,99].

Epigallocatechin gallate (EGCG), the main polyphenol in green tea, seems to have an important antioxidant action, countering the ROS generation and exerting a powerful scavenger of free radicals in periodontitis [100]. At the same time, ECGC stimulates the antioxidant defences. A study conducted by Hrishi et al. has highlighted how the daily use of a green tea-based toothpaste should increase the activity of glutathione-S-transferase in the gingival crevicular fluid of patients with CPD [101].

Genistein, the main isoflavone in soy, appears to be a powerful antioxidant and an inhibitor of nitric oxide synthesis. A study has shown how injections of 20 mg/kg of body weight of genistein, in mouse models with periodontitis, protect cells from mitochondrial damage and the ROS accumulation, as well as reduce the inflammatory cytokine levels in periodontal tissue [102].

Another NBC with antioxidant action useful in countering the progression of periodontal disease is lycopene, a carotenoid mainly contained in tomatoes. In vivo studies showed how oral lycopene supplementation stimulates the physiological antioxidant defences. Moreover, these studies highlight its possible use as adjuvant treatment in the management of periodontal disease and of gingivitis [103,104].

Numerous animal studies suggest that resveratrol, the main polyphenol of red wine, is able to counteract the onset and the progression of periodontal disease, thanks to its powerful antioxidant, as well as anti-inflammatory actions [105]. In particular, resveratrol seems to stimulate antioxidant enzymes activity, such as superoxide dismutase, catalase, and peroxidase [106].

### 4.4. Anticancer Effects

Among the NBCs, polyphenols seem to have an important anticancer action, both through systemic mechanisms, such as the OS and chronic inflammation reduction, and through topical mechanisms, such as the induction of cellular apoptosis and the inhibition of growth, invasion, and metastasis of cancer cells [107,108]. Therefore, the polyphenols could be a new potential adjuvant treatment for some types of cancer, including oral squamous cell carcinoma (Figure 2) [109].

A recent in vitro study investigated the possible effect of green tea EGCG in slowing growth of cancer cells, in particular of oral cancer cell lines H400 and H357. The authors pointed out how cell growth and migration capacity of both cell lines were deeply slowed down after treatment with EGCG extracts [110]. Interestingly, it has been shown that the oral intake of green tea EGCG seems to be able to reduce the nephrotoxic effect due to the use of cisplatin, one of the most common chemotherapy drugs used in the treatment of bladder, neck, lung, and testicular cancer [111]. In fact, cisplatin seems to act specifically in the kidney, stimulating mitochondrial OS, exerting nephrotoxic effects, which can lead to acute kidney damage or CKD worsening [112,113,114]. Therefore, the use of green tea polyphenols through both systemic and topical administration could represent a valid adjuvant strategy to counteract the progression of oral cancers and at the same time protect against the nephrotoxicity of common anticancer drugs.

### 4.5. Other Beneficial Effects

NBCs are able to slow down the alveolar bone resorption process that can be observed in patients with CKD-MBD, as demonstrated by several studies (Figure 2) [115].

The local administration of extracts of Ginkgo biloba leaves, an ancient tree belonging to the Ginkgoaceae family, seems to inhibit the alveolar bone loss that is observed in periodontitis [116]. In fact, these extracts stimulate osteoblasts to produce bone alkaline phosphatase, an enzyme that plays a key role in the bone mineralization process [117].

*Rhinacanthus nasutus* L., a natural herb of Chinese origin rich in carotenoids, appears to exert an anticlastogenic action, by inhibiting the receptor activator of nuclear factor-kB ligand (RANKL) action, a factor produced by osteoblasts under various stimuli, including inflammatory ones, responsible for the differentiation and the activation of osteoclasts [118]. These data suggest how this plant could be used in the treatment of osteoporotic process, even to the alveolar bone.

Propolis, a natural resin produced by bees, seems to protect the alveolar bone tissue from demineralization induced by periodontitis [119]. This NBC inhibits the production and the activation of osteoclasts, and it stimulates the production and the activation of osteoblasts in the alveolar bone [120].

## 5. Conclusions

Extensive scientific evidence suggest that oral health is closely related to individual general health. In fact, the presence of an oral disease could lead to an increased risk of CDNCDs onset, such as CKD. A mutual cause–effect relationship exists between oral disease and CKD and the possible link is represented by systemic chronic inflammation and oral microbiota dysbiosis. In this context, the use of NBCs capable of exercising, through different mechanisms, important beneficial effects in the prevention and in the treatment of oral diseases represents a valid strategy to counteract the onset and progression of CKD and its comorbidities, as suggested by several scientific evidence collected in this review.

In this perspective, the formulation of a new mouthwash/gel/gingival paste with a high content of polyphenols derived from the minor polar compounds of extra virgin olive oil [121], in association with NBCs with antimicrobial action, such as tannins from *Castanea sativa* [122], could represent a new therapeutic tool for oral diseases in CKD patients. In particular, for uremic stomatitis, a gel based on aloe vera and clove oil could be formulated. The former would appear to reduce pain and inflammation, while the latter is able to relieve pain.

## Data Availability

Not applicable.

## Abbreviations

8-OHdG	8-hydroxydeoxyguanosine
CCL28	Chemokine ligand 28
CDNCDs	Chronic non-communicable degenerative diseases
CKD	Chronic kidney disease
CKD-MBD	Chronic kidney disease- mineral and bone disorder
CMV	Cytomegalovirus
COX	Cyclooxygenase
CPD	Chronic periodontitis disease
EGCG	Epigallocatechin gallate
ESRD	End stage renal disease
GO	Gingival overgrowth
HD	Hemodialysis
HSV	Herpes virus simplex
ICAM	Intercellular adhesion molecule
IL	Interleukin
MMPs	Matrix metalloproteinases
NBCs	Natural bioactive compounds
NF-kB	Factor-κB
OS	Oxidative stress
PEW	Protein-Energy Wasting
RANKL	Receptor activator of nuclear factor-kB ligand
ROS	Reactive oxygen species
RRT	Renal replacement therapy
TNF	Tumor necrosis factor
VCAM	Vascular cell adhesion molecule
